# Controlling the Wake-Up Mechanism and Switching Kinetics of Ferroelectric Hf_*x*_Zr_1 – *x*_O_2_ through Hf Content Modulation

**DOI:** 10.1021/acsami.5c15572

**Published:** 2025-10-29

**Authors:** Athira Sunil, Ruben Alcala, Cláudia Silva, Thomas Mikolajick, Suzanne Lancaster

**Affiliations:** † 138823 NaMLab gGmbH, Dresden 01187, Germany; ‡ Chair of Nanoelectronics, TU Dresden, Dresden 01187, Germany

**Keywords:** ferroelectrics, Hf content, wake-up, phase transition, ferroelastic switching, switching kinetics, domain nucleation, domain propagation

## Abstract

The excellent scalability and compatibility to current CMOS manufacturing processes make ferroelectric Hf_*x*_Zr_1 –_ *_x_* O_2_ thin films a promising candidate for embedded nonvolatile memories, as well as for synaptic devices in neuro-inspired computing. In order to achieve precise control over the polarization states and to ensure reliable operation in these thin films, a thorough understanding of the film’s domain switching kinetics and behavior under field cycling is necessary. The Hf composition in Hf_*x*_Zr_1 – *x*_O_2_ thin films plays a crucial role in determining the disorders, phase composition, and crystallographic texture within the film when integrated in a metal–ferroelectric–metal (MFM) device, all of which affect the evolution of its field cycling response and switching kinetics. In this work, the impact of Hf content on wake-up and domain switching kinetics in Hf_*x*_Zr_1 – *x*_O_2_ thin films is investigated, and the physical mechanisms behind these differences are explored. This study highlights that multiple wake-up mechanisms can coexist in the same film and that as the Hf composition is increased, the dominant physical mechanism of wake-up in the film changes from a field-induced phase transition to field-induced ferroelastic domain switching. Furthermore, since the inhomogeneity within the ferroelectric film depends on Hf composition, the speed of polarization switching and the available partial polarization states in the film can be precisely controlled as a function of Hf content due to the disorder-driven domain nucleation and nonlinear domain wall dynamics.

## Graphical abstract

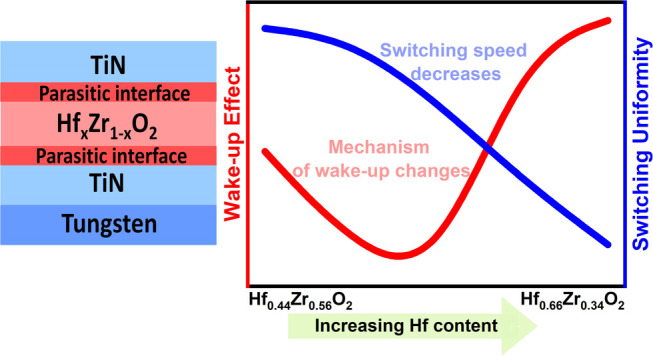

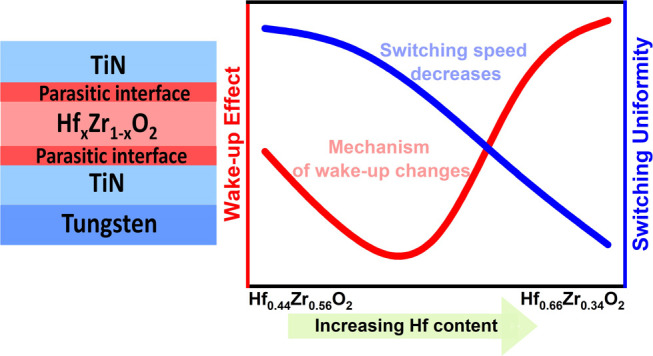

## Introduction

Fast and energy-efficient computing paradigms are necessary in the current era of data-driven applications, such as Artificial Intelligence (AI) and the Internet of Things (IoT). The structural separation of processing and memory devices in conventional computing architectures poses a great challenge, referred to as the von Neumann bottleneck, due to a mismatch between the logic and memory speed as well as high energy costs.^1^ Brain-inspired neuromorphic computing systems, where information is simultaneously processed and stored to memory, have gained substantial popularity as the next-generation computing architecture due to their potential to offer great parallelism and plasticity for energy-efficient processing and computing.^2^ Embedded nonvolatile memories (eNVMs) with programmable conductance are considered a potential candidate to efficiently emulate the functionalities of a biological nervous system, like synaptic plasticity and neuronal activities.^3^ Ferroelectric memory devices offer significant advantages over flash or other emerging eNVM devices in terms of fast switching and power consumption efficiency due to their purely field-driven switching mechanism.^4^

The discovery of ferroelectricity in fluorite-structured hafnium oxide (HfO_2_)^5^ spurred great research interest in the material due to its unique advantages over conventional perovskite-structured ferroelectric materials, namely excellent compatibility with current complementary-metal-oxide–semiconductor (CMOS) manufacturing processes and stable ferroelectricity at the nanometer scale,^6^ demonstrating promising potential to solve the challenge of high-density device integration. Nonetheless, these ferroelectric films based on HfO_2_ suffer from several performance instabilities that hinder their commercial implementation. One of the main challenges associated with HfO_2_-based ferroelectric thin films is the increase in remanent polarization (commonly defined as 2P_*r*_) with field cycling, referred to as the “wake-up effect”.^7^ The wake-up effect is a critical issue that jeopardizes the reliability of HfO_2_-based memory devices. A varying 2P_*r*_ with cycling modifies the memory window and introduces uncertainty, particularly during multilevel operation, where the polarization of each state needs to be precisely and reproducibly controlled. Several hypotheses have been proposed to explain the origin of the wake-up effect. These include (i) crystal structure changes, i.e., phase transition from the nonpolar centrosymmetric tetragonal (t) or monoclinic (m) phase to polar noncentrosymmetric orthorhombic (o) phase;^8,9^ (ii) depinning of ferroelectric domains due to a decrease in built-in bias as a result of oxygen vacancy redistribution;^10^ and (iii) irreversible ferroelastic domain switching of the in-plane oriented polar axis to the out-of-plane direction.^11^ Nevertheless, no single universal origin of wake-up has been demonstrated, and as we will show here, each mechanism leads to a distinct signature that impacts device reliability in a different way. Another factor that governs the performance of HfO_2_-based memory devices is the domain switching dynamics during polarization reversal, which involves nucleation of oppositely polarized domains followed by domain wall propagation.^12^ Since HfO_2_ generally follows nucleation-limited switching (NLS) dynamics,^13^ the activation barrier for domain nucleation is typically considered the critical factor, although the speed of subsequent domain propagation may also play a crucial role in defining the uniformity of switching and the macroscopic coercive field (*E*_c_) of the HfO_2_-based ferroelectric film.^14^ Therefore, it is necessary to understand the underlying mechanisms controlling the ferroelectric behavior of HfO_2_-based thin films for reliable device operation.

Numerous research efforts have been made to stabilize the ferroelectric polar orthorhombic phase in HfO_2_-based thin films, commonly by forming alloys of hafnium and zirconium oxides (HZO)^15^ or by doping HfO_2_ with various dopants, including Si, La, and Y, among others.^16–18^ Alloyed Hf_*x*_Zr_1 – *x*_O_2_ is the most widely researched since the ferroelectric o-phase can be formed at a lower thermal budget and with a wide range of Hf/Zr concentrations.^15,19^ Modifying the Hf content has been proposed as a route to stabilizing Hf_*x*_Zr_1 – *x*_O_2_ at reduced thicknesses^6^ by circumventing the tendency of thinner Hf_0.5_Zr_0.5_O_2_ films to crystallize in the tetragonal phase^20^ since the phase composition and crystallization temperatures depend sensitively on Zr content.^21^ However, the influence of Hf content on the ferroelectric switchability or wake-up effect is rarely studied.^22^ The elemental composition of Hf_*x*_Zr_1 – *x*_O_2_ alloys affects the residual mechanical stress,^23^ crystal phase composition,^15,21^ concentration of oxygen vacancies, and thereby the passive dead layer thickness,^24^ all of which control the film’s ferroelectric behavior. Therefore, the choice of Hf_*x*_Zr_1 – *x*_O_2_ alloy composition could play an important role in engineering the wake-up and switchability of the ferroelectric film.

In this study, we elucidate the role of Hf content in the wake-up mechanism and polarization switching kinetics of Hf_*x*_Zr_1 – *x*_O_2_ thin films. Combined with structural analysis, endurance, and switching kinetics measurements, a comparative study on the wake-up and polarization switching mechanism was carried out as a function of Hf content at a fixed film thickness of 8 nm. Characterization of the wake-up effect at varying electric fields revealed that different wake-up mechanisms can coexist in a given film and that the dominant physical mechanism of wake-up changes as a function of phase composition, which can be primarily linked to the crystallographic texture. Moreover, the observed wake-up mechanisms show different characteristic dependencies on the magnitude of the applied field. The analysis of switching kinetics further demonstrates that the domain evolution with the field strongly depends on the phase composition. This work provides a more comprehensive understanding for the material design strategies of ferroelectric Hf_*x*_Zr_1 – *x*_O_2_ thin films.

## Results and Discussion

### Structural Analysis

Multiple crystallographic phases with the same chemical formula, termed polymorphs, can coexist in fluorite-structured ferroelectric Hf_*x*_Zr_1 – *x*_O_2_ thin films. These include the ferroelectric orthorhombic (o) phase (space group: Pca2_1_), antiferroelectric tetragonal (t) phase (space group: *P*4_2_/*nmc*), and paraelectric monoclinic (m) phase (space group: *P*2_1_/*c*). Figure 1a shows the Grazing Incidence X-ray Diffraction (GIXRD) patterns of the 8 nm thick Hf_*x*_Zr_1 – *x*_O_2_ films with the Hf content varying from *x* = 0.44 to *x* = 0.66. The GIXRD pattern verifies polymorphism in all the films, and the peaks at 28.5°, 30.5°, 31.7°, and 35.6° correspond to m(−111), o(111)/t(011), m(111), and o{200}/t(002) planes, respectively. The combination of the orthorhombic (200), (020), and (002) planes is collectively represented as o{200}. To better visualize the observed differences, zoomed-in plots of the areas of interest with overlaid GIXRD patterns of Hf_*x*_Zr_1 – *x*_O_2_ films are shown in the inset. As the Hf content is increased to *x* = 0.60, small diffraction peaks from the (−111) and (111) planes of the monoclinic phase appear as shoulders to the diffraction peak from the (111)/(011) planes of the orthorhombic/tetragonal phases. However, due to the limitation in the resolution of the diffraction data presented, further structural and electrical analyses are necessary to confirm the presence of the monoclinic phase in these films. Also, a shift in the position of the o(111)/t(011) peak to lower 2θ is observed, which could be correlated to the reduction of the tetragonal phase in films with higher Hf content due to stress relaxation. However, the deconvolution of tetragonal and orthorhombic phases cannot be reliably carried out from the GIXRD peak alone due to similar lattice parameters. One interesting observation is that the intensity of the peak at 35.6°, attributed to the {200}-oriented orthorhombic phase, increased with increasing Hf content. To better perceive this observed difference in intensity, the integrated area under the peak at 35.6° was normalized to the integrated area of the peak from the (111) plane of TiN at 36.6°, and the results are shown in Figure 1b. This difference in the relative peak area from the {200} planes of the orthorhombic phase can be interpreted as a difference in the preferred crystallographic texture of the Hf_*x*_Zr_1 – *x*_O_2_ thin films as the Hf content is varied. Moreover, an additional diffraction peak corresponding to the (110) plane of the orthorhombic phase emerges for Hf_*x*_Zr_1 – *x*_O_2_ thin films with higher Hf content (*x* = 0.60, 0.66), further adding to the disperse crystallographic texture in these films. One possible origin for the difference in the crystallographic texture of the Hf_*x*_Zr_1 – *x*_O_2_ films with varying Hf content is the differences in the interface between the bottom TiN electrode and the Hf_*x*_Zr_1 – *x*_O_2_ film. The thickness of the parasitic TiO_*x*_ bottom interface layer is reported to reduce with the incorporation of HfO_2_ in the Hf_*x*_Zr_1 – *x*_O_2_ film due to the higher formation energies of oxygen vacancies and interstitials in HfO_2_ compared to ZrO_2_.^24,25^ A thicker TiO_*x*_ bottom interface layer prevents texture transfer from the bottom TiN electrode to the Hf_*x*_Zr_1 – *x*_O_2_ film and promotes the growth of {111} planes, which are energetically favorable due to their lower surface energy compared to {200} and {110} planes. Therefore, a higher fraction of {200}- and {110}-oriented orthorhombic planes would imply an improved texture transfer from the {200} and {220} planes of the underlying TiN bottom electrode,^26,27^ which is consistent with an expected lower interfacial layer thickness. Additionally, it was experimentally shown that these {200} planes are preferentially oriented parallel to the Hf_*x*_Zr_1 – *x*_O_2_ film surface.^28^

**Figure 1:**
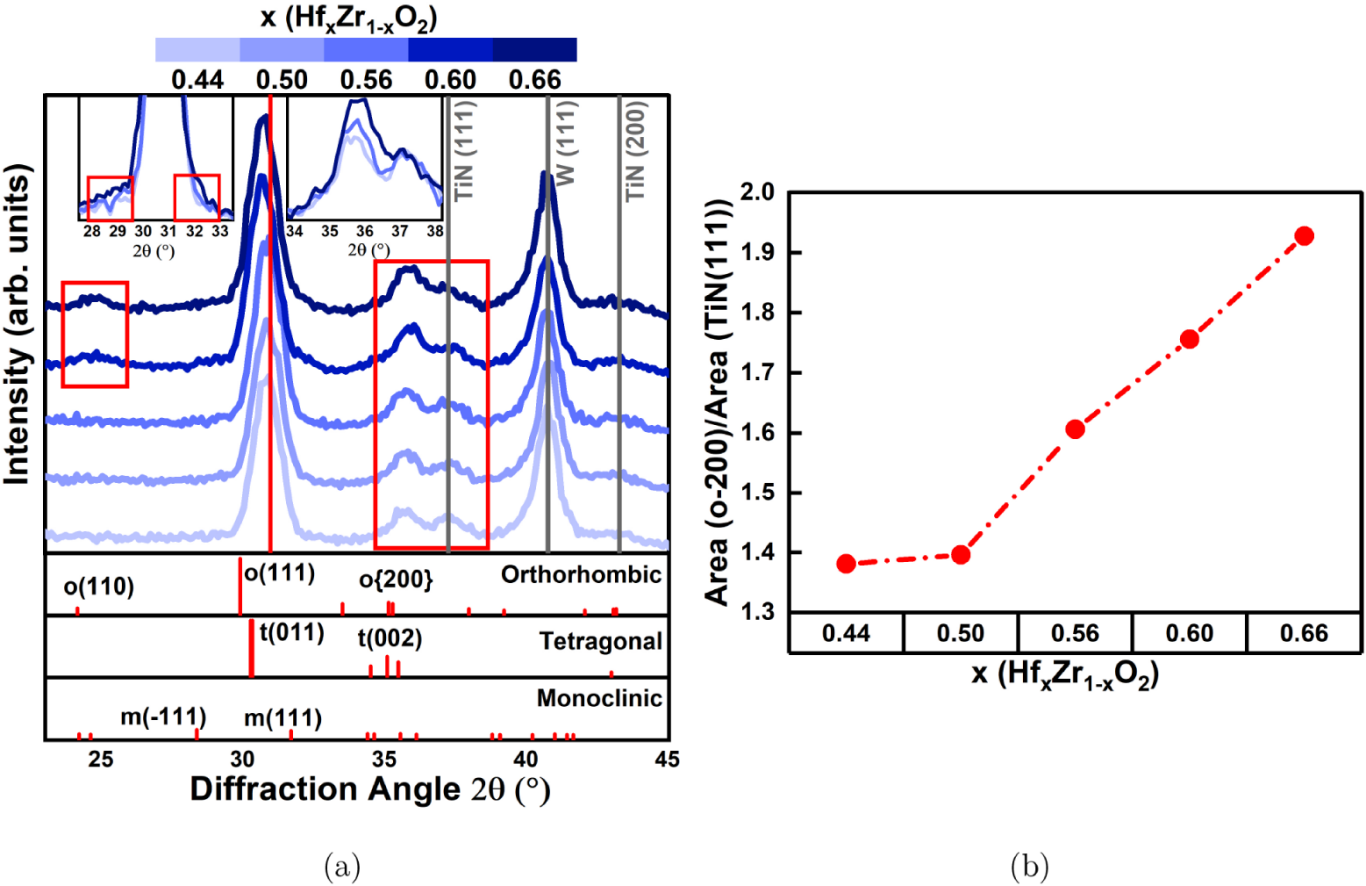
a) GIXRD patterns of MFM structures for various Hf_*x*_Zr_1 – *x*_O_2_ compositions; the insets show the overlaid GIXRD patterns of MFM structures to visualize the intensity difference of the diffraction peaks from the monoclinic phase fraction (∼28.5° and 31.7°) and the {200}-oriented orthorhombic phase fraction (∼35.6°) for various Hf_*x*_Zr_1 – *x*_O_2_ compositions, and b) the integrated area of the peak from the {200} orientation of the orthorhombic phase at 35.6° normalized to the integrated area of the peak from the (111) plane of TiN at 36.6° as a function of the Hf content of the film.

To gain further insight into the structural composition, the biaxial in-plane tensile strain is determined according to the sin^2^ψ method from the Bragg–Brentano XRD measurements. A stronger in-plane tensile strain favors the tetragonal phase, while a weaker in-plane tensile strain stabilizes the monoclinic phase.^23^ From Figure 2, it can be observed that the in-plane tensile strain decreases as the Hf content is increased from *x* = 0.44 to *x* = 0.66, which could be attributed to the phase composition in the film changing from a mixture of tetragonal and orthorhombic phases to a mixture of orthorhombic and monoclinic phases, as supported by the GIXRD patterns.

**Figure 2:**
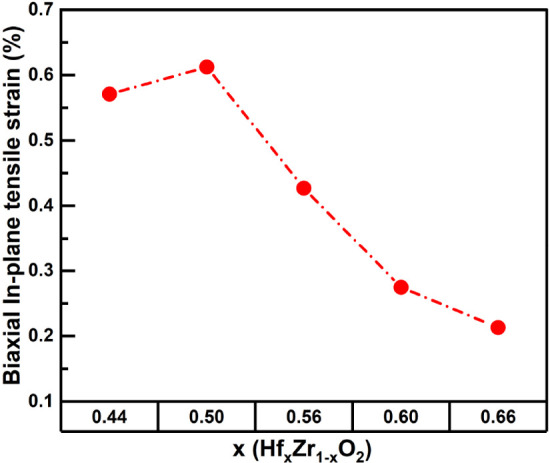
In-plane tensile strain of Hf_*x*_Zr_1 – *x*_O_2_ films as a function of the Hf content analyzed by using Bragg–Brentano XRD and the sin^2^ψ method.

### Electrical Analysis

To further support the inferences from the structural analyses of these Hf_*x*_Zr_1 – *x*_O_2_ films with various Hf compositions, capacitance versus electric field (*C*–E) measurements were performed on the MFM capacitors to obtain the dielectric permittivity of these films. The *C*–E measurements were performed with a DC bias sweep between −4 MV/cm and 4 MV/cm and a 50 mV amplitude sinusoidal signal at a 10 kHz frequency. The antiferroelectric tetragonal phase is reported to have the highest dielectric permittivity, followed by the ferroelectric orthorhombic phase, and the paraelectric monoclinic phase has the least dielectric permittivity.^15^ Here, the capacitance value at 4 MV/cm, away from the peaks in the *C*–E curve, is used to calculate a reliable dielectric permittivity value without domain wall contributions to the capacitance. Figure 3 depicts the average dielectric permittivity of Hf_*x*_Zr_1 – *x*_O_2_ films measured in the pristine state and after 10^4^ electric field cycles. In the pristine state, films with lower Hf content show a reduced dielectric permittivity, again evidencing the presence of a thicker parasitic TiO_*x*_ bottom interface layer. A low-permittivity interfacial layer would reduce the overall measured permittivity, while the presence of the high-k tetragonal phase has been confirmed in these films from their higher in-plane tensile strain. An even more significant decrease in dielectric permittivity values is seen in Hf_*x*_Zr_1 – *x*_O_2_ films with higher Hf content (*x* = 0.60, 0.66), corroborating the evidence from GIXRD and strain analyses that the incorporation of higher Hf content results in the inclusion of a monoclinic phase fraction in Hf_*x*_Zr_1 – *x*_O_2_ films. Moreover, after 10^4^ electric field cycles, it is observed that the change in the dielectric permittivity as compared to the pristine state is less significant in Hf_*x*_Zr_1 – *x*_O_2_ films with higher Hf content (*x* = 0.60, 0.66) as compared to Hf_*x*_Zr_1 – *x*_O_2_ films with lower Hf content, indicating that a change in the phase composition with electric field cycling is occurring in Hf_*x*_Zr_1 – *x*_O_2_ films with lower Hf content and not in those with higher Hf content.

**Figure 3:**
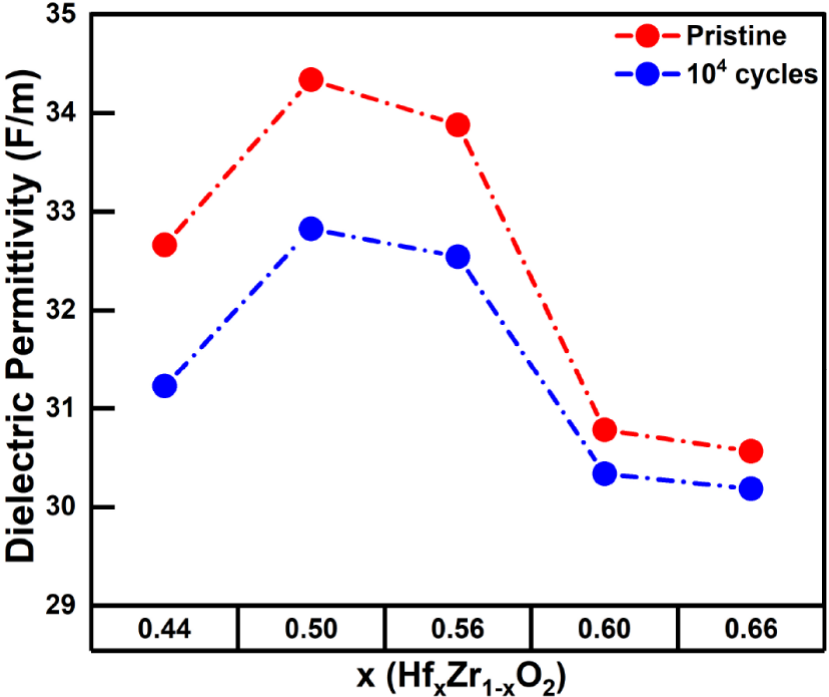
Dielectric permittivity of MFM capacitors in the pristine state and after 10^4^ bipolar electric field cycles of amplitude 4 MV/cm and 10 kHz frequency for various Hf_*x*_Zr_1 – *x*_O_2_ compositions.

Figure 4a and b illustrates the current density versus electric field (*J*–*E*) and polarization versus electric field (*P*–*E*) hysteresis loops, respectively, of Hf_*x*_Zr_1 – *x*_O_2_ films measured in the pristine state and after 10^4^ electric field cycles. Here, 10^4^ electric field cycles were sufficient to wake up all the films, and negligible increase in polarization was observed with further electric field cycles (illustrated in Figure S1). The electric field cycling was carried out using trapezoidal bipolar pulses of a field amplitude of 4 MV/cm and 10 kHz frequency. The dynamic hysteresis currents were measured in response to triangular pulses of amplitude 4 MV/cm and frequency 10 kHz, and the polarization hysteresis loop was reconstructed by integrating the displacement current response. All the films exhibit an antiferroelectric-like pinched hysteresis loop in the pristine state, corresponding to forward and back-switching peaks in *J*–*E* hysteresis, which merge with electric field cycling. As the Hf content is increased from *x* = 0.44 to *x* = 0.56, the pinching in the pristine state is suppressed, and the forward switching field, as depicted in Figure 5a, is reduced from 2.4 MV/cm to 1.8 MV/cm. After wake-up, the hysteresis loops open up completely, and a sharp switching peak is observed. However, with a further increase of the Hf content to *x* = 0.60, a difference in the overall electrical behavior of the Hf_*x*_Zr_1 – *x*_O_2_ film is observed. The forward switching field increases significantly to 3.5 MV/cm, and the overall switching contribution is reduced, which can be observed from the difference in the switchable polarization (spontaneous polarization) attained in these films in the pristine state and after wake-up. After wake-up, two partially merged current peaks can be observed in the *J*–*E* loops, a sharp switching peak at lower fields and a broader peak at higher fields, making the overall switching peak broader and asymmetric. It should be noted that these two current peaks do not merge any further with additional electric field cycling (see Figure S2) and the broader switching peak at higher fields has been associated with the presence of the (110) oriented orthorhombic phase in previous reports.^29^ Figure 5a also illustrates the coercive field of Hf_*x*_Zr_1 – *x*_O_2_ with various Hf contents, and it can be observed that the average coercive field, which was extracted at zero polarization, steadily increases with increasing Hf content. In order to verify that the increased coercive field at higher Hf content is not just an artifact of their broader switching peak distribution, given the only partially merged additional switching current peak, the field for maximum ferroelectric switching is also illustrated in Figure 5a. This again shows a steady increase with an increasing Hf content. Moreover, it can be clearly observed that the variation in the coercive field after wake-up and the forward switching field in the pristine state for different Hf compositions cannot be correlated.

**Figure 4:**
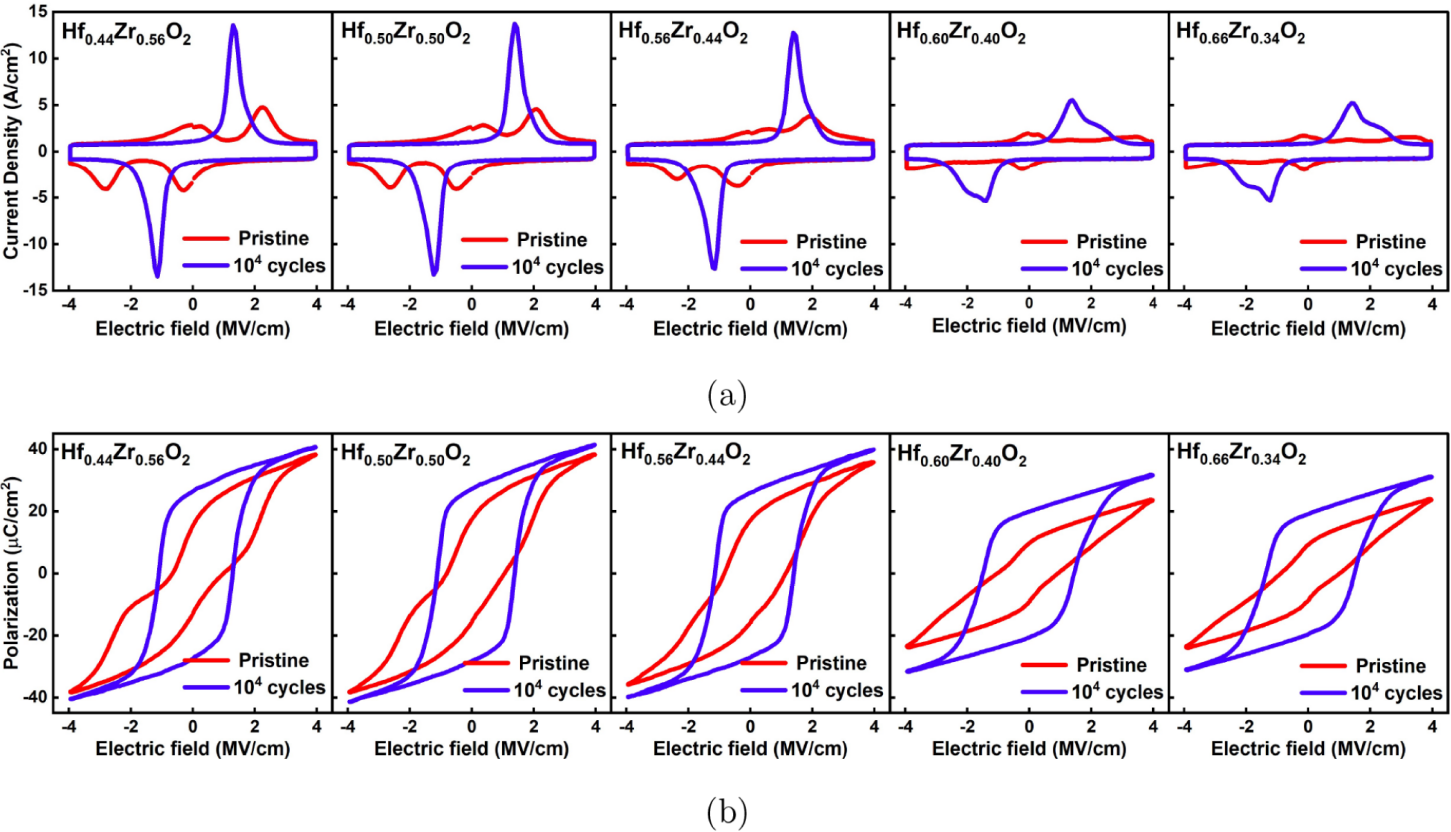
a) Current density vs electric field and b) corresponding polarization vs electric field characteristics of MFM capacitors in the pristine state and after 10^4^ bipolar electric field cycles of amplitude 4 MV/cm and 10 kHz frequency for various Hf_*x*_Zr_1 – *x*_O_2_ compositions.

**Figure 5:**
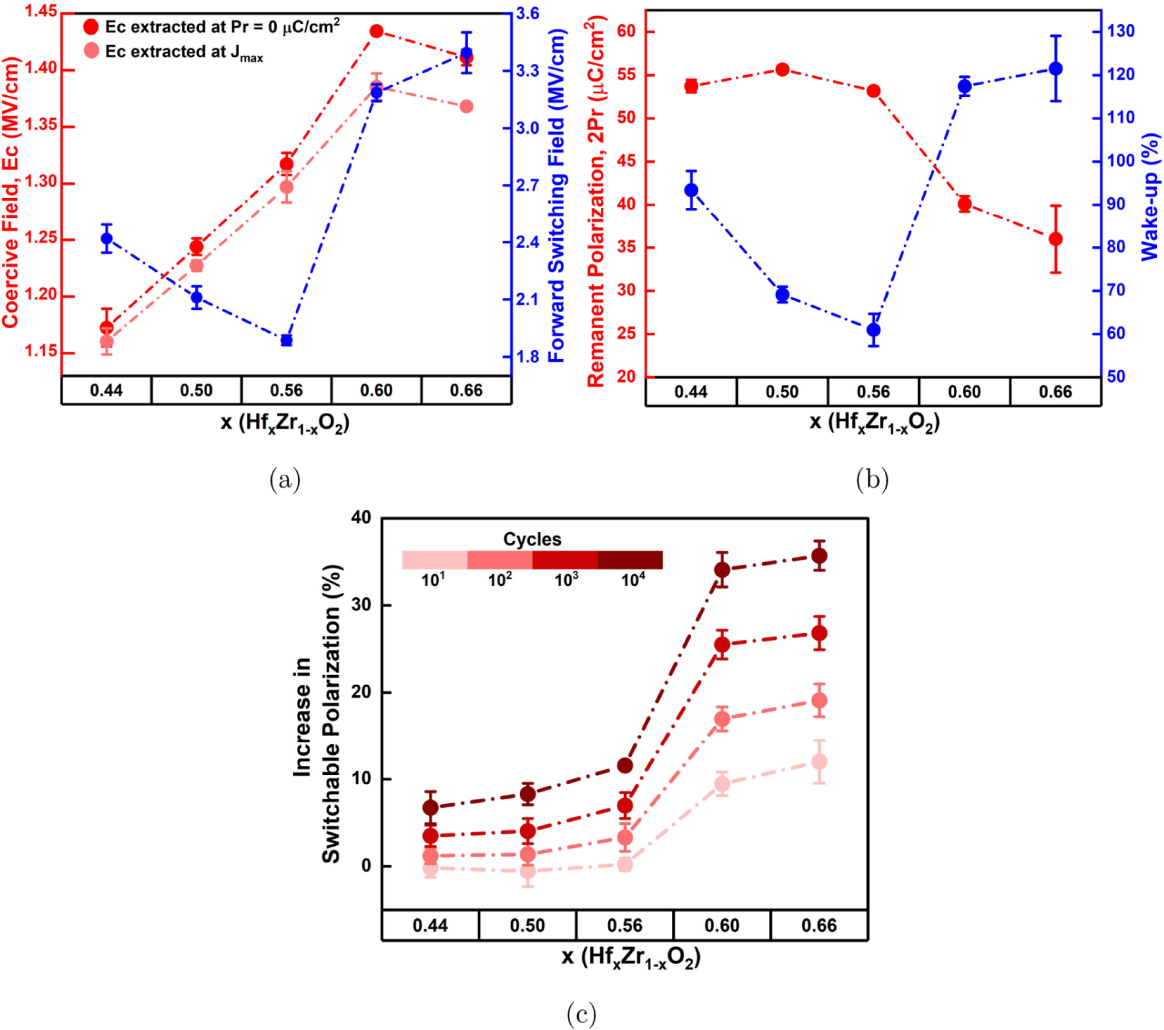
a) Coercive fields after wake-up (red curves; left axis) and forward switching fields in the pristine state (blue curves; right axis), b) remanent polarization after wake-up (red curves; left axis) and wake-up effect (blue curves; right axis), measured at an electric field amplitude of 4 MV/cm, and c) variation of switchable polarization attained after bipolar electric field cycling at 4 MV/cm, as a function of the Hf content in the MFM capacitors.

From Figure 5b, it can be observed that the remanent polarization value after wake-up, which was extracted at the zero external electric field, decreases with Hf content incorporation. This effect could be ascribed to the presence of the monoclinic phase in these films as backed up from the previous structural and electrical analyses and has also been reported in previous literature.^15^ The wake-up effect is defined here as the percentage increase in remanent polarization after 10^4^ electric field cycles:

1 $$\mathrm{Wake-up}=\left(\frac{2P_{r}\left(10^{4}\mathrm{cycles}\right)-2P_{r}\left(\mathrm{Pristine}\right)}{2P_{r}\left(\mathrm{Pristine}\right)}\right)\times 100$$

As illustrated in Figure 5b, the wake-up effect decreases initially when increasing the Hf content from *x* = 0.44 to *x* = 0.56 and is followed by a sharp increase as the Hf content is further increased to *x* = 0.60. This trend in wake-up could be directly correlated to the observed behavior in the forward switching field of these films in the pristine state (i.e., a higher forward switching field leads to a larger wake-up effect). One possible cause for the higher forward switching field in the pristine state is high in-plane stress in the Hf_*x*_Zr_1 – *x*_O_2_ thin films.^30^ However, in our case, the stress steadily decreases for films with higher Hf content. Therefore, the cause for the higher forward switching field observed in the pristine state for films with both lower (*x* = 0.44) and higher (*x* = 0.60, 0.66) Hf content should essentially have different roots. Next, in Figure 5c, we compare the switchable polarization, that is, the polarization value extracted from *P*–*E* loops at 4 MV/cm so that the pinching of the hysteresis loop does not affect the analysis. The increase compared to that of the pristine state is plotted for up to 10^4^ electric field cycles. It is interesting to note that the difference in saturation polarization attained in the pristine state and after wake-up significantly increases with increasing Hf content. A 30% increase is observed in the saturation polarization attained as the Hf content is increased from *x* = 0.44 to *x* = 0.66. The negligible increase in the saturation polarization after wake-up in low Hf content films indicates that the wake-up process involves the stabilization of a reversible transition from a nonpolar to polar state during switching, with little evidence of additional domains contributing to the switching process with electric field cycling. However, the considerable increase in saturation polarization after wake-up at high Hf contents suggests an irreversible transition to a polar state with each cycling field and that additional domains are taking part in the switching process with cycling, which is suggestive of either a nonpolar-to-polar phase change or domain reorientation. The pristine *J*–*E* curves as well as results from the structural analysis rule out a significant tetragonal phase fraction in the pristine state, and the monoclinic-to-polar orthorhombic phase transition is energetically unfavorable,^31^ which can also be evidenced from the dielectric permittivity of these films before and after wake-up, as shown in Figure 3. Thus, the observed differences insinuate that the mechanism of wake-up is different in films with lower and higher Hf content or that the dominant mechanism of wake-up changes in Hf_*x*_Zr_1 – *x*_O_2_ thin films as the composition is varied.

To better assess the difference in wake-up mechanisms, Hf_*x*_Zr_1 – *x*_O_2_ thin films with various Hf content were cycled at varying electric fields, and the percentage of wake-up observed after 10^4^ electric field cycles is illustrated in Figure 6. The wake-up is calculated as defined in eq 1 in the previous section. A strong field dependence of wake-up as a function of Hf composition is observed, where the field at which the wake-up is most prominent shifts to higher values with increasing Hf content in the Hf_*x*_Zr_1 – *x*_O_2_ thin films, indicating a higher energy barrier for wake-up in the films as the Hf content is increased. For films with lower Hf content, the wake-up effect is most pronounced at fields as low as 1.5 MV/cm, and with further increases of the electric field amplitude, it decreases exponentially until reaching a minimum value. However, for films with higher Hf content, the wake-up starts only at fields above 2 MV/cm, and it increases steadily as the magnitude of the electric field is increased. In other words, with Hf content higher than *x* = 0.60 in the Hf_*x*_Zr_1 – *x*_O_2_ films, cycling at fields as high as 2 MV/cm is required to transition from paraelectric to ferroelectric behavior. These observations serve as evidence of the initial hypothesis that the prominent mechanism of wake-up is dependent on the composition of the Hf_*x*_Zr_1 – *x*_O_2_ thin films. In addition, it is interesting to note that the Hf_0.56_Zr_0.44_O_2_ films do not show a pronounced wake-up effect in any of the applied electric fields. The clear difference in behavior at low and high fields, and the transition between behaviors as Hf content is increased, indicates that multiple wake-up mechanisms coexist in the films and that the dominant mechanism is impacted both by the Hf composition and by the magnitude of the applied field during cycling.

**Figure 6:**
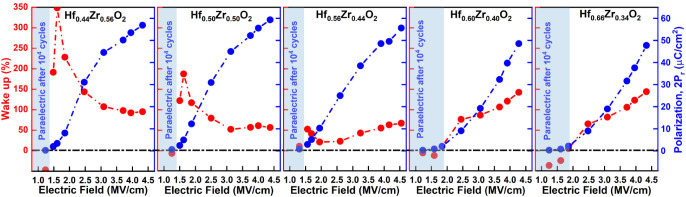
Wake-up effect as defined by eq 1 (red curves; left axis) and remanent polarization (blue curves; right axis) vs electric field of MFM capacitors for various Hf_*x*_Zr_1 – *x*_O_2_ compositions.

The low field wake-up effect of Hf_*x*_Zr_1 – *x*_O_2_ thin films with lower Hf content is attributed to the tetragonal to orthorhombic phase transition due to the low transition barrier of only 30 meV/f.u, which is related to the soft phonon mode interpolating between the tetragonal and orthorhombic phases.^31–33^ The presence of a tetragonal phase fraction in these films in the pristine state is supported by the higher in-plane residual stress and the observed strongly pinched hysteresis loop in these films. Reyes-Lillo et al. calculated a critical field strength of 1.2 MV/cm necessary to overcome this barrier of 30 meV/f.u.^32^ This calculated field strength is in good agreement with our experimental results, where a large wake-up effect is observed at electric field amplitudes of 1.2–1.5 MV/cm. The slightly higher field requirement is most likely due to a parasitic voltage drop due to the presence of interfaces in these films. Materlik et al. argues that the relaxation of the structure back to the tetragonal phase at the removal of the external electric field is due to this low phase transition barrier that can be overcome by thermal energy alone, while with cycling the orthorhombic phase is irreversibly stabilized over the tetragonal phase.^31^ The presence of an antipolar orthorhombic phase (space group: *Pbca*) as an intermediate state in the phase transition pathway from nonpolar tetragonal to polar orthorhombic phase cannot be excluded; however, the presence of antipolar orthorhombic phase in the pristine state is not plausible since the phase transition from antipolar to polar orthorhombic phase generally involves negligible wake-up,^34,35^ which is not the case here. To explain the dominant mechanism behind the high-field wake-up effect observed in Hf_*x*_Zr_1 – *x*_O_2_ thin films with a higher Hf content, the ferroelastic switching of the in-plane oriented {200} polar axis to the out-of-plane direction under the application of an external electric field is considered. The presence of higher fraction of {200} oriented domains and the large difference in the proportion of switchable polarization in these films before and after wake-up, as observed from the GIXRD and electrical analyses, respectively, support the idea that the domains with {200} crystal orientation undergo reorientation from in-plane to out-of-plane direction during electric field cycling. The higher barrier to ferroelastically switch the in-plane oriented {200} domains to out-of-plane direction, as evidenced from the persistent paraelectric behavior during cycling at field magnitudes up to 2 MV/cm and the steadily increasing wake-up effect with a further increase of the electric field amplitudes, can be explained from the stronger mechanical constraint from the substrate on the {200} textured domains.^28,36^ Hence, switching of {200} textured domains is more constrained by the substrate than that for the {111} textured domains. Finally, the overall reduced wake-up effect at all fields in the Hf_0.56_Zr_0.44_O_2_ films can be explained as follows: 1) the residual in-plane stress in these films is within the range to mainly stabilize orthorhombic phase. The absence of a high fraction of the tetragonal phase in these films in the pristine state can be observed from the partially merged current peaks and lower forward switching field. Hence, the wake-up effect involving tetragonal to orthorhombic phase transition at lower amplitudes of electric fields is less pronounced. 2) The fraction of {200} oriented domains in these films is lower when compared to the films with higher Hf content (*x* ≥ 0.60). As a result, the observed wake-up effect due to ferroelastic switching in these films at higher electric fields is lower. Nonetheless, a coexistence of the two wake-up mechanisms and a transition in the driving mechanism of wake-up is observed as a small increase in wake-up when the amplitude of field cycling is increased. It should also be noted that depinning of the ferroelectric domains during electric field cycling can be considered as a coeffect of both wake-up mechanisms involving field-induced phase transition and field-induced ferroelastic switching. Pinning of the domains could be induced by a built-in field as a consequence of various disorders, like oxygen vacancies, charged defects, and boundaries due to dispersed crystallographic textures and phase compositions, all of which act as domain pinning sites.^37,38^ In the case of Hf_*x*_Zr_1 – *x*_O_2_ films with lower Hf content, since the tetragonal phase is stabilized by oxygen vacancies, it is defect-rich compared to the orthorhombic phase, and the electric field-induced phase transition from the tetragonal to orthorhombic phase could result in oxygen vacancy redistribution and hence domain depinning due to the reduction of the built-in field.^9,39^ In the case of Hf_*x*_Zr_1 – *x*_O_2_ films with higher Hf content, the 90° reorientation of the polar axis due to field-induced ferroelastic switching results in a more uniform distribution of the domains, consequently resulting in domain depinning by reducing the pinning sites. Therefore, the presence or coexistence of domain depinning during electric field cycling cannot be excluded. However, in the context of our studies, it cannot be considered as the physical mechanism that distinguishes the observed differences in wake-up processes in Hf_*x*_Zr_1 – *x*_O_2_ thin films as a function of the Hf composition.

Next, the influence of Hf content on the polarization switching kinetics of the Hf_*x*_Zr_1 – *x*_O_2_ thin films is investigated since they directly correlate to the macroscopic coercive field of the film and, hence, its switchability.^40,41^ As discussed previously and shown in Figure 5a and b, the observed wake-up behavior in Hf_*x*_Zr_1 – *x*_O_2_ thin films could not be directly correlated to their coercive fields but rather to the forward switching field in the pristine state, while the macroscopic coercive field of a woken-up device tends to increase with Hf content. In other words, wake-up is dominated by the energy barrier for phase transition or domain reorientation, but the macroscopic coercive field describes the switching of all domains in the system, which can have additional influences including local field inhomogeneities, crystal phases, or domain nucleation energies. Figure 7a shows the normalized switched polarization as a function of switching pulse amplitude and duration for awakened Hf_*x*_Zr_1 – *x*_O_2_ films with various Hf contents. Initially, a 4.2 MV/cm, 500 μs pulse was applied to polarize the dipoles in the same direction. Then, the amplitude (0.25–4 MV/cm) and the width (20 ns–1 s) of the write pulse is varied, and a fixed read voltage pulse (4.2 MV/cm, 500 μs) is used to obtain the amount of switched polarization using positive-up-negative-down (PUND) measurements. The black solid lines are obtained by fitting the experimental data to the nucleation limited switching model.^13^ Unlike bulk epitaxial ferroelectric films which follow the Kolmogorov–Avrami–Ishibashi (KAI) model of switching, where the sideway expansion of the domains after nucleation is unrestricted,^42^ in polycrystalline films, a domain cannot propagate indefinitely due to their higher impurity concentration and the presence of grain boundaries. Instead, switching is better explained by the nucleation-limited model, which assumes that the film has many heterogeneous nucleation sites with independent switching kinetics.^13^ Accordingly, the time-dependent normalized switched polarization, Δ*P*_norm_, can be expressed by assuming a Lorentzian distribution of the logarithm of the characteristic switching time:^38^

2 $$\Delta P_{\mathrm{norm}}=\frac{\Delta P\left(t\right)}{2P_{s}}=\int_{-\infty }^{\infty }\left[1-e^{-{\left(t/t_{0}\right)}^{n}}\right]F(\log\left(t_{0}\right)\times d\left(\log\left(t_{0}\right)\right)dx$$

with

3 $$F(\log\left(t_{0}\right)=\frac{A}{\pi }\left[\frac{w}{((\log\left(t_{0}\right)-{\left(log(t_{1})\right)}^{2}+w^{2}}\right]$$

where *A*, *w,* and log­(*t*_1_) are the normalization constant, half width at half-maximum, and central value of the Lorentzian distribution of the logarithm of the characteristic switching time *F*(log­(*t*_0_)). Jo et.al. associated this Lorentzian distribution of *F*(log­(*t*_0_)) with the presence of a local field *E* with a Lorentzian distribution $F\left(E\right)=\frac{A}{\pi }\left[\frac{\Gamma }{E^{2}+\Gamma^{2}}\right]$ at the ferroelectric domain pinning sites.^38^ Here, Γ is the half width at half-maximum and represents the inhomogeneity in the local field distribution. The domain growth at a low external field region is governed by a thermal activation process at the pinning sites,^43^ and Jo et.al. proposed that with the effect of local field on domain wall growth, the associated *t*_0_ can be expressed such that the distribution in local fields results in a distribution of log­(*t*_0_), with $\log\left(t_{1}\right)\approx E_{a}\left(\frac{1}{E_{ext}}\right)$ and $w\approx \Gamma E_{a}\left(\frac{1}{E_{\mathrm{ext}}^{2}}\right)$ , where *E*_a_ is the activation field.^38^ The inverse proportionality between the characteristic switching time and external electric field is also in accordance with Merz’s empirical equation, $t_{1}=t_{0}e^{E_{a}/E_{\mathrm{ext}}}$ .^44^ A detailed derivation can be found elsewhere.^38,44^ The origin of Merz’s law is ascribed as nucleation of a reversed domain or, analogously, as creep behavior of the ferroelectric domain walls in a pinning potential, where thermally activated hopping induces domain wall movement from one local minimum to the next.^45,46^ Accordingly, the distribution of independently nucleating domains and domain wall creep motion can be associated with the spatially inhomogeneous distribution of local switching fields, which could be due to impurities, crystal defects, or the intrinsic inhomogeneity of the ferroelectric film.^47^ Therefore, the inhomogeneity in the local electric field can be used as an effective tool to elucidate the mechanism behind the observed kinetics of polarization reversal.^37,48–50^ Although the activation barrier for domain nucleation is considered the critical factor in the NLS model of polarization reversal and the lateral domain wall movement is neglected, it is reasonable to assume that domain propagation becomes energetically favorable after a critical nucleus size is reached.^51,52^ Hence, at sufficiently large electric field amplitudes, the speed of domain propagation may also play a crucial role in determining the uniformity in switching.^14,47^

**Figure 7:**
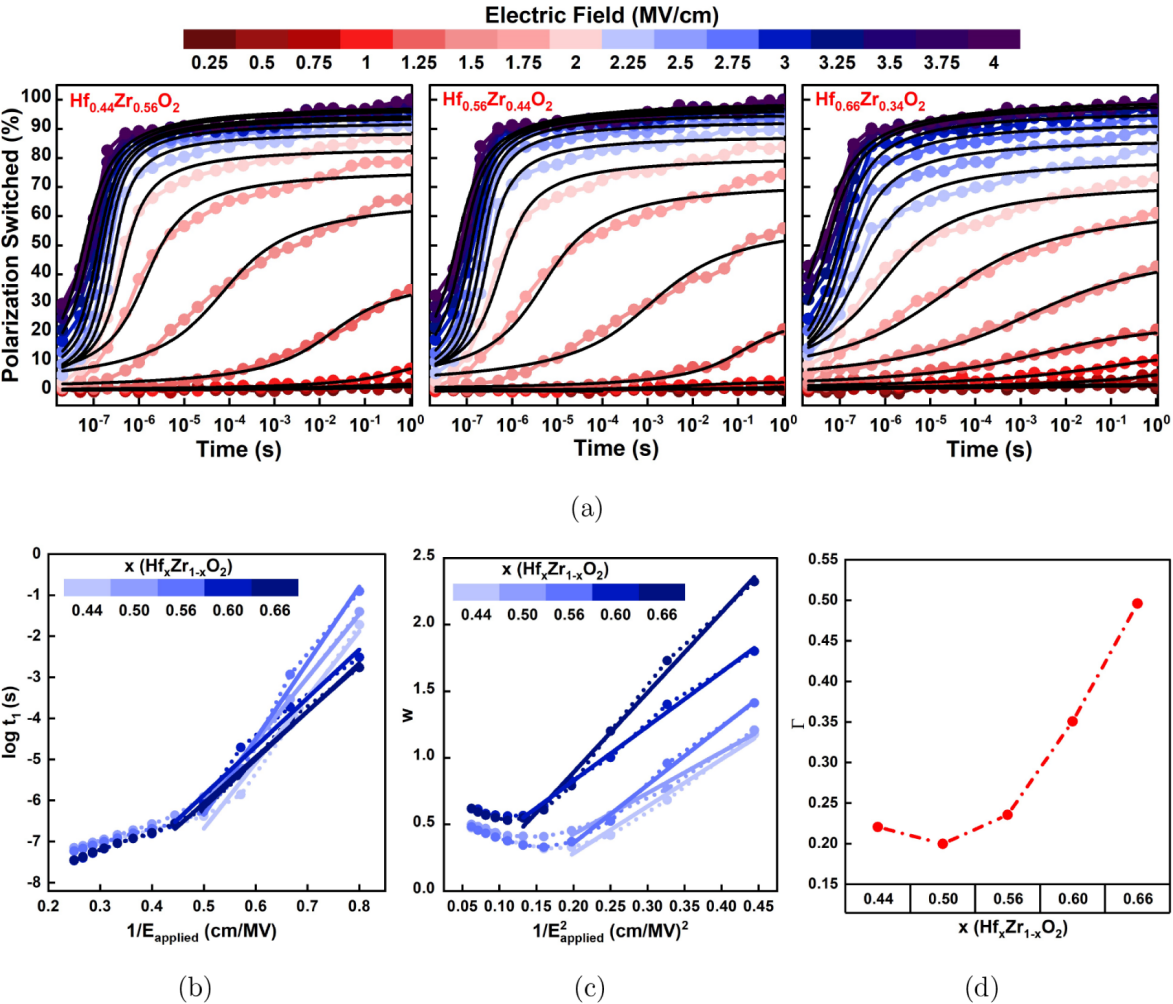
a) Switched polarization as a function of pulse width and pulse amplitude (switching kinetics) for Hf_*x*_Zr_1 – *x*_O_2_ thin films with three different Hf compositions (*x* = 0.44, *x* = 0.56, and *x* = 0.66); external field dependence on the b) central value (log­(*t*_1_)). c) Half-width at half-maximum (*w*) of Lorentzian distribution of the logarithm of characteristic switching time; d) half-width at half-maximum (Γ), representing the inhomogeneity in the local field distribution, for various Hf_*x*_Zr_1 – *x*_O_2_ compositions.

For the Hf compositions considered in this study, it can be observed from Figure 7a that at lower field amplitudes (*E*_ext_ < *E*_c_), the onset of switching is fastest for films with higher Hf content (Hf_0.66_Zr_0.34_O_2_), followed by films with lowest Hf content (Hf_0.44_Zr_0.56_O_2_), and the Hf_0.56_Zr_0.44_O_2_ film switches the slowest. At intermediate field amplitudes (*E*_c_ < *E*_ext_ < 2*E*_c_), the normalized switched polarization is the lowest for films with higher Hf content, i.e., the switching is slower. This can be visualized as a decrease in the slope of the kinetic switching curves of the Hf_0.66_Zr_0.34_O_2_ films corresponding to field amplitudes comparable to its coercive field. The slope of the polarization switching characteristics can be attributed to the domain propagation effect, and a decrease in the slope of the kinetic switching curves for field amplitudes close to the coercive field indicates the need to consider the mechanism of propagation of domain walls in addition to nucleation of the reversed domains to analyze the polarization switching. At higher field amplitudes (*E*_ext_ ≥ 2*E*_c_), higher Hf content films again switch more easily at short pulse widths (≤100 ns). However, as the pulse duration is increased, an abrupt shift in the proportion of reversed polarization to ≥ 90% is observed for all the films which then saturates for further increase of the pulse duration. Figure 7b and c illustrates the relationship between log­(*t*_1_) versus 1/*E*_ext_ and *w* versus $1/E_{ext}^{2}$ , respectively for Hf_*x*_Zr_1 – *x*_O_2_ films with various Hf contents. At low external fields, the inverse linear proportionality is met for both log­(*t*_1_) and *w*. The slope of the linear fit in Figure 7b at low field magnitudes represents the activation field *E*_a_, while the slope of the linear fit in Figure 7c represents the concentration of pinning sites that hinder domain propagation during polarization reversal. Figure 7d illustrates the inhomogeneity in the local electric field, represented by the half width at half-maximum of the local field distribution at ferroelectric domain pinning sites, for Hf_*x*_Zr_1 – *x*_O_2_ films with varying Hf composition and is extracted from the slopes of the linear fits from Figure 7b and c. The observed difference in the onset of switching in Hf_*x*_Zr_1 – *x*_O_2_ films with various Hf content (Figure 7a) at low applied fields can be explained from the difference in the activation fields of these films. Hf_*x*_Zr_1 – *x*_O_2_ films with Hf content of *x* = 0.56 have the highest activation field, followed by films with Hf content of *x* = 0.44 and films with Hf content of *x* = 0.66 have the lowest activation field. Hence, the slower onset of switching can be directly attributed to the higher activation field. Since the domain reversal at low magnitudes of applied electric field is mainly nucleation limited, the lower activation field for polarization reversal in the low field regime suggests the presence of a larger number of nucleation sites.^53^ The observed increase in polarization switched under short pulses of larger field magnitudes in Hf_0.66_Zr_0.44_O_2_ films can also be attributed to the presence of more nucleation sites, which suggests that to facilitate domain propagation during polarization reversal, the applied external pulse should have sufficiently long pulse duration in addition to its large magnitude. Various factors including oxygen vacancies, charged defects, interface defects, interfacial dead layer, different orientations of the ferroelectric domains, and also competing nonferroelectric phases can act as nucleation sites in ferroelectric Hf_*x*_Zr_1 – *x*_O_2_ films.^7,49,54,55^ In the case of films with lower Hf content, the following disorders could aid in the reduction of the energy barrier for the nucleation of domains with reverse orientation: 1) thicker interfacial dead layer and thereby more interface defect sites and 2) higher fraction of oxygen vacancies in the bulk of the film due to their redistribution as a result of tetragonal to orthorhombic phase transition during wake-up. The effect of competing nonferroelectric layers and domain orientations in these films can be neglected since the tetragonal phase that was present in these films in the pristine state transforms to the ferroelectric orthorhombic phase after the wake-up process and the orientation of the ferroelectric domains in these films is more uniform when compared to films with higher Hf content. In the films with higher Hf content, a more disperse distribution of domain orientations and the presence of nonferroelectric monoclinic phase in addition to the ferroelectric orthorhombic phase in the film, which is reported to result in denser domain boundaries,^14^ could be the disorders aiding in faster nucleation and thus a lower activation energy. In the case of Hf_0.56_Zr_0.44_O_2_ films, the lower density of the above-mentioned disorders when compared to the other films results in a higher barrier for nucleation for polarization reversal. These disorders, however, not only aid in lowering the energy barrier for nucleation of the domains for polarization reversal but also induce inhomogeneity in the local electric field in the film due to dipole defects at the domain pinning sites. This results in a complicated landscape for domain wall propagation and induces pinning-driven nonlinear domain wall dynamics, namely the domain wall creep.^14,56^ The linear inverse proportionality of log­(*t*_1_) and *w* with *E*_ext_ is indicative of the domain wall creep regime^38^ and is met for lower to intermediate external field magnitudes (<2*E*_c_). However, with higher applied field magnitudes (≥2*E*_c_), the linear proportionality is no longer fulfilled and the switching time and local fluctuations in domain dynamics related to the concentration of pinning sites reach a minimum average value implying a faster and more uniform switching. As observed from Figure 7b and c, for films with higher Hf content (*x* = 0.60, 0.66), this linear inverse proportionality of log­(*t*_1_) and *w* with *E*_ext_ is persistent to higher field magnitudes as compared with films of lower Hf content, indicating hindered domain wall movement even under higher field magnitudes, which directly leads to a lower switched polarization. This can be explained from the high local field inhomogeneity for these films, as evidenced in Figure 7d, where it is observed that the inhomogeneity in the local field distribution increases drastically as the Hf content is increased to *x* = 0.60 due to the higher concentration of domain pinning sites in these films, consistent with previous reports.^48^ This suggests that either the presence of denser domain boundaries due to nonuniform phase distribution or more disperse domain distribution in the films with higher Hf content has a bigger influence on inducing the inhomogeneity in the field distribution in the Hf_*x*_Zr_1 – *x*_O_2_ films, rather than the other defects observed in the films with lower Hf content. Nonetheless, the impact of oxygen vacancies or interfacial defects on the field inhomogeneity can be observed in that the Hf_0.50_Zr_0.50_O_2_ film has the narrowest local field distribution, while decreasing the Hf content to *x* = 0.44 already shows a slight increase in the inhomogeneity of the local field. Since the coercive field of the Hf_*x*_Zr_1 – *x*_O_2_ films, extracted from the displacement current response under a bipolar triangular pulse of 4 MV/cm amplitude and 50 μs duration, displays a steady increase with increasing Hf content, it can be concluded that, in addition to nucleation of the heterogeneous domains, the impact of domain propagation during polarization reversal cannot be fully neglected and the higher coercive field in Hf_*x*_Zr_1 – *x*_O_2_ films with higher Hf content can be attributed to a restricted domain propagation due to a more disperse phase composition and domain orientation. These results suggest a route for engineering the switching speed and width of the switching peak at varying applied fields through modulating the hafnium content, where both the speed^57^ and the width of the switching peak^58^ affect the behavior of Hf_*x*_Zr_1 – *x*_O_2_ films in nonvolatile, multilevel ferroelectric devices.

## Conclusion

The influence of the Hf content on the wake-up effect and switching kinetics in 8 nm thick Hf_*x*_Zr_1 – *x*_O_2_ films was investigated. Control over the wake-up effect and domain switching kinetics is vital to ensure the reliability and reproducibility of the polarization states necessary to achieve multilevel capability. This work showed that multiple wake-up mechanisms can coexist in the same film, and the dominant physical mechanism behind the wake-up effect changes from a field-induced phase transition to field-induced ferroelastic domain switching as a function of Hf composition, which can be related to the phase composition and crystallographic texture of the film. Additionally, the analysis of polarization switching kinetics revealed that the domain evolution with field magnitude and duration strongly depends on the disorders and inhomogeneities in the ferroelectric film, which again varies as a function of Hf composition due to the differences in phase composition, texture, and electrode interface. These observations demonstrate that engineering phase composition and crystallographic texture in Hf_*x*_Zr_1 – *x*_O_2_ films by modulating the Hf content can be used as an effective route to control the wake-up mechanism and the kinetics of ferroelectric polarization reversal.

## Experimental Section

Sample fabrication: the metal–ferroelectric–metal (MFM) stack was deposited on a p-doped silicon (*p*-Si) substrate. The bottom electrode composed of 25 nm tungsten (W) followed by 10 nm titanium nitride (TiN) deposited at room temperature (RT) using direct current (DC) sputtering in a Bestec Ultrahigh Vacuum System. The ferroelectric layer was then deposited at 280 °C using atomic layer deposition (ALD) in an Oxford OpAL system. The metal–organic precursors HyALD­(HfCp­(NMe_2_)_3_) and ZyALD (ZrCp­(NMe_2_)_3_) from Air Liquide were used. Ozone was used for the oxidation steps during the process. The Hf composition (*x* = 0.44, 0.50, 0.56, 0.60, and 0.66) was varied by adjusting the HfO_2_:ZrO_2_ ALD cycling ratio (4:5, 1:1, 5:4, 3:2, and 2:1) during deposition. The nominal values of *x* are used, which assume equal growth rates of HfO_2_ and ZrO_2_. The top electrode composed of 10 nm TiN deposited at RT using direct current (DC) sputtering in a Bestec Ultrahigh Vacuum System. Rapid thermal annealing (RTA) at 440 °C for 240 s was performed to crystallize the Hf_*x*_Zr_1 – *x*_O_2_ films in a N_2_ atmosphere after the top deposition. Further, the samples were annealed at 400 °C for 1 h to mimic the impact of the back-end of line thermal budget on real devices. To structure the capacitors, the following steps were performed: (1) lithography using flood exposure, (2) deposition of a 5 nm thick titanium adhesion layer and 40 nm thick platinum contact films by electron-beam evaporation using a Bestec GmbH tool, (3) liftoff in acetone and an ultrasonic bath, and (4) inductively coupled plasma etching of the TiN top electrode using a Plasmalab System133 from Oxford Instruments.

Characterization: GIXRD and BBXRD were performed in a Bruker D8 Discover tool with a 0.154 nm wavelength of Cu–Kα radiation. GIXRD and BBXRD were performed on samples after processing, i.e., after annealing, top contact (Ti/Pt) deposition, and etching of the top TiN layer. The measured capacitor area was approximately 900 μm^2^. Electrical measurements were performed using Keithley 4225 pulse measurement units controlled with a Keithley 4200A-SCS parameter analyzer on a Cascade Microtech probe station and a TF Analyzer 3000 instrument (aixACCT Systems). Voltage pulses were applied to the top electrode, while the bottom electrode was grounded in all measurements. The contact to the bottom electrode was achieved through electrical breakdown of one capacitor.

## Supplementary Material



## Notes

Bipolar electrical field cycling of Hf_*x*_Zr_1 – *x*_O_2_ capacitors­(Section S1); remanent polarization vs field cycles of MFM capacitors for various Hf_*x*_Zr_1 – *x*_O_2_ compositions (Figure S1); and current density vs field of MFM capacitors with varying bipolar electric field cycles of amplitude 4 MV/cm and 10 kHz frequency for various Hf_*x*_Zr_1 – *x*_O_2_ compositions (Figure S2) (PDF)

## #

CIC nanoGUNE, Tolosa Hiribidea 76, 20018 Donostia/San Sebastian
