# Supplementary material for: Controlling the Wake-Up Mechanism and Switching Kinetics of Ferroelectric Hf x Zr1 – x O2 through Hf Content Modulation
Source: ACS Appl Mater Interfaces. 2025 Oct 29;17(45):62708–19. doi: 10.1021/acsami.5c15572 (PMC12616602; doi:10.1021/acsami.5c15572)
Supplement: Supplementary file 1 [file am5c15572_si_002.pdf]

Supporting Information
Controlling the wake-up mechanism and switching kinetics of ferroelectric $Hf_xZr_{1-x}O_2$ through Hf content modulation

Athira Sunil,[*,†,‡] Ruben Alcala,[†] Cláudia Silva,[†] Thomas Mikolajick,[†,‡] and Suzanne Lancaster[†,¶]

†*NaMLab gGmbH, 01187 Dresden, Germany*

‡*TU Dresden, 01187 Dresden, Germany*

¶*Current address: CIC nanoGUNE, Tolosa Hiribidea 76, 20018 Donostia / San Sebastian*

E-mail: athira.sunil@namlab.com

Section S1: Bipolar electrical field cycling of $Hf_xZr_{1-x}O_2$ capacitors

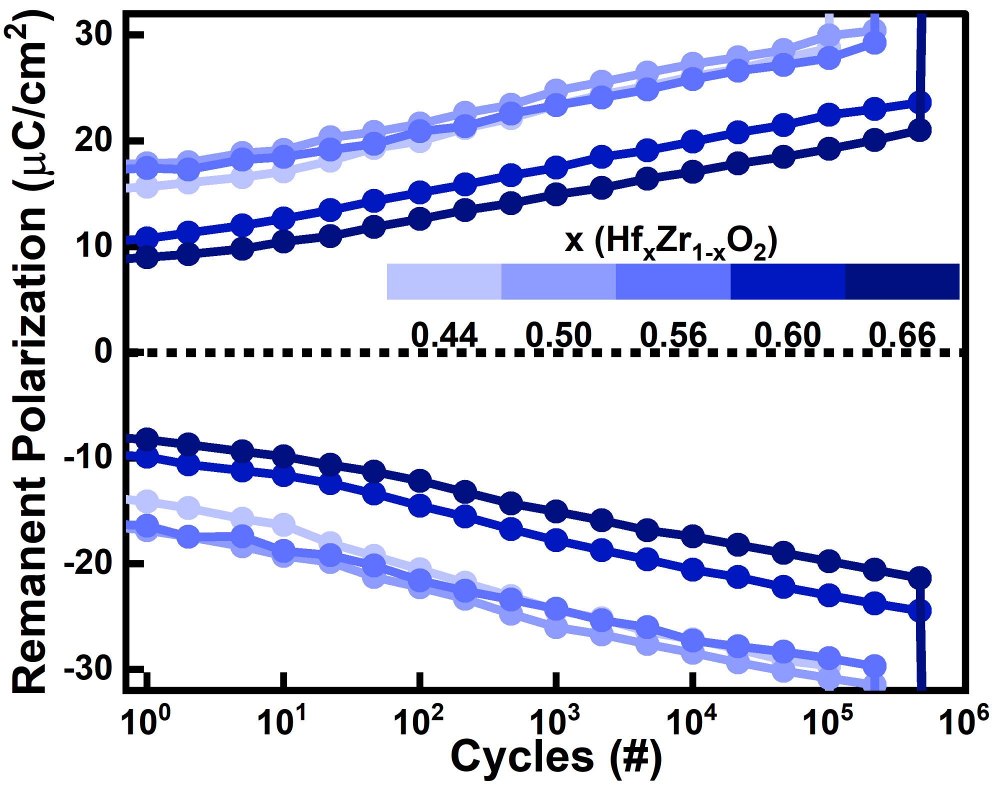

Figure S1: Remanent polarization vs. field cycles (4 MV/cm, 10 kHz) of MFM capacitors for various $Hf_xZr_{1-x}O_2$ compositions

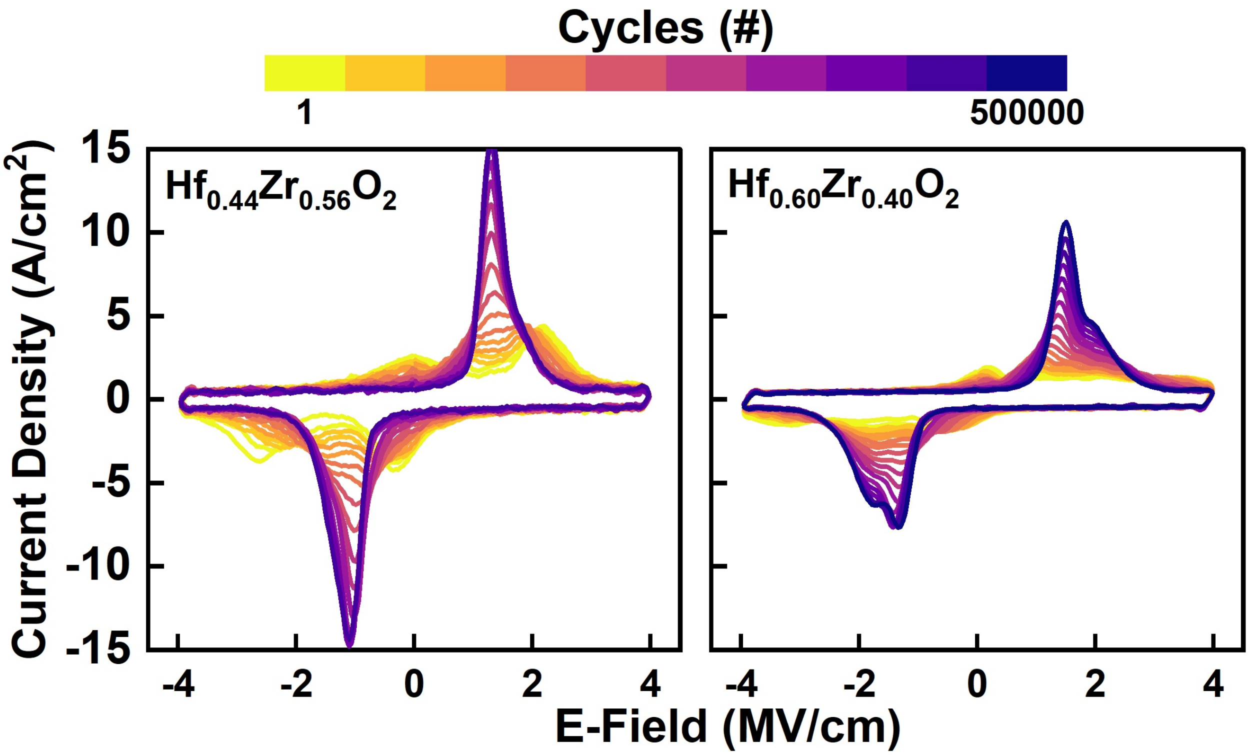

Figure S2: Current density vs. field of MFM capacitors with varying bipolar electric field cycles of amplitude 4 MV/cm and 10 kHz frequency, for various $Hf_xZr_{1-x}O_2$ compositions.
